# Restoring integrity—A grounded theory of coping with a fast track surgery programme

**DOI:** 10.3402/qhw.v11.29864

**Published:** 2016-01-08

**Authors:** Lene Bastrup Jørgensen, Bengt Fridlund

**Affiliations:** 1 Centre of Elective Surgery, Aarhus University, Aarhus C, Denmark; 2 Interdisciplinary Research Unit, Regional Hospital Silkeborg, Silkeborg, Denmark; 3 School of Health Sciences, Jönköping University, Jönköping, Sweden

**Keywords:** Coping preferences, self-management, fast track programme, orthopaedic surgery, grounded theory, integrity, education level, self-reported health status

## Abstract

**Aims and objectives:**

The aim of this study was to generate a theory conceptualizing and explaining behavioural processes involved in coping in order to identify the predominant coping types and coping type-specific features.

**Background:**

Patients undergoing fast track procedures do not experience a higher risk of complications, readmission, or mortality. However, such programmes presuppose an increasing degree of patient involvement, placing high educational, physical, and mental demands on the patients. There is a lack of knowledge about how patients understand and cope with fast track programmes.

**Design:**

The study design used classical grounded theory.

**Methods:**

The study used a multimodal approach with qualitative and quantitative data sets from 14 patients.

**Results:**

Four predominant types of coping, with distinct physiological, cognitive, affective, and psychosocial features, existed among patients going through a fast track total hip replacement programme. These patients’ main concern was to restore their physical and psychosocial integrity, which had been compromised by reduced function and mobility in daily life. To restore integrity they economized their mental resources, while striving to fulfil the expectations of the fast track programme. This goal was achieved by being mentally proactive and physically active. Three out of the four predominant types of coping matched the expectations expressed in the fast track programme. The non-matching behaviour was seen among the most nervous patients, who claimed the right to diverge from the programme.

**Conclusion:**

In theory, four predominant types of coping with distinct physiological, cognitive, affective, and psychosocial features occur among patients going through a fast track total hip arthroplasty programme.

Fast track regimens presuppose patient involvement (Norlyk & Harder, 2009, 2010) and make demands on the patient's mental and physical capacity. A fast track total hip arthroplasty (THA) programme has the pivotal element of motivating patients to be active and attuned to the expectations featured in the fast track programme: “Patients should be informed and motivated to be active patients and their expectations should be modulated in order to improve satisfaction” (Husted, Jensen, Solgaard, & Kehlet, 2012, p. 1). In line with this expected health behaviour, the focus on patient responsibilities and their role in managing and coping with altered health conditions has grown substantially, with an increasing focus on health policy. Supporting patients in meeting these demands is key to the desired transition of health behaviour (WHO, 2013). We lack knowledge about the psychological and behavioural profiles of patients undergoing THA (Husted, Solgaard, Hansen, Soballe, & Kehlet, 2010), including coping behaviour and coping capacity. This study aims to present a theory on how patients predominantly cope with a fast track THA programme.

## Background

Osteoarthritis is a frequent reason for hip complaints, including pain and functional disability, and is the most common indication for THA (Woolf et al., 2003), which is one of the most successful surgical treatments (Learmonth, Young, & Rorabeck, 2007). In 2012, 8787 patients underwent THA in Denmark, 79% of which were grounded in primary (idiopathic) arthrosis (Danish Register of Hip Arthroplasty, 2013). The number of patients suffering from primary arthrosis, and therefore in need of THA, is expected to increase due to the consequences of obesity, increased physical activity, and an expectation of a high quality of life among elderly people (Changulani, Kalairajah, Peel, & Field, 2008).

A fast track THA programme is an accelerated intervention and joint recovery programme comprising a multidisciplinary and multimodal intervention and clinical pathway focusing on preoperative optimization and patient education, reducing the surgical stress response, optimizing pain relief, and enforcing mobilization and nutritional support (Husted et al., 2010). Development of less invasive surgical procedures and better regimens for handling post-operative pain have made it possible to develop fast track orthopaedic procedures for THA, thereby reducing hospitalization time (Andersen, Pfeiffer-Jensen, Haraldsted, & Soballe, 2007; Larsen, Hvass, Hansen, Thomsen, & Soballe, 2008). Consequently, fast track methodologies have reduced the length of stay (LOS) significantly when applied to total hip replacement (Pivec, Johnson, Mears, & Mont, 2012).

Patients undergoing fast track procedures do not experience a higher risk of complications, readmission, or mortality (Husted et al., 2010). However, an increasing degree of patient involvement is prerequisite (Norlyk & Harder, 2009, 2010), placing educational, physical, and psychological demands on the patients. Empowering patients to meet these demands is crucial (WHO, 2013). The concept of patient empowerment is widely recognized as providing patients with opportunities and the environment to develop the skills, confidence, and knowledge to move from being a passive recipient of care to an active partner in their healthcare (Lancet Editorial, 2005). Empowering patients to go through surgical procedures is a multidimensional process influenced by psychological, behavioural, and educational components. Healthcare professionals need to take these components into consideration when involving patients in the treatment and management of their recovery. We know that coping is a multidimensional phenomenon (Jørgensen, Dahl, Pedersen, & Lomborg, 2013a, 2013b; Jørgensen, Lomborg, Dahl, & Pedersen, 2014; Skinner, Edge, Altman & Sherwood, 2003) that has been well-described in chronically ill patients compared to patients in fast track orthopaedic programmes. We lack knowledge on how patients cope with a fast track THA programme. The aim of this study was to generate a theory conceptualizing and explaining behavioural processes involved in coping in order to identify the predominant coping types and coping type-specific features. The identification of coping preferences may form the basis for offering patients support to meet the demands of a fast track programme based on their individual counselling needs.

## Methods

### Design

Classical grounded theory (GT) was chosen as the overall design (Glaser, 1978; Glaser & Strauss, 1967). The study followed Glaser's advice for generating a theory with the potential to explain patterns of behaviour (Glaser, 1978). Thus, the task was to carefully type behaviour, not people (Glaser, 1978).

Coping behaviour is a complex and multidimensional phenomenon (Skinner, Edge, Altman & Sherwood, 2003) with physiological, cognitive, affective, and psychosocial dimensions (Jørgensen et al., 2013b, 2014). Furthermore, the educational, physiological, and psychological demands of the fast track THA called for combining both quantitative and qualitative data. To match this complexity, a grounded theory approach holding multiple data collection and analysis processes was chosen with the intention of capturing and explaining the interacting dimensions of coping behaviour. This multimodal approach (Jørgensen et al., 2013a) is in line with classical GT, which creates the opportunity to combine qualitative and quantitative data to generate a theory grounded in data from the empirical area under study (Glaser, 2008; Glaser & Strauss, 1967).

### The theoretical framework

A grounded theory transcends description and gives a theoretical explanation for social processes in the empirical world (Glaser, 1978). Consequently, a limiting feature of our study is that it cannot provide full and accurate explanations; instead, it focuses on core behaviour with the potential to offer an explanation for the main concern of the people in question (Glaser, 1978). Generation of a GT has three pivotal elements: a joint data collection and analysis process, theoretical sampling, and writing memos. This was done throughout a three-phased, open, selective, and theoretical procedure (Glaser, 1978; Glaser & Strauss, 1967).

#### Theoretical sampling

Theoretical sampling was the procedure by which we jointly collected and analysed data and decided the data to collect next and where in order to develop the theory as it emerged (Glaser & Strauss, 1967). Thus, theoretical sampling constituted an inductive and iterative process that abstained from using preconceived concepts as a theoretical framework (Glaser & Strauss, 1967).

Data hold latent patterns, and data can be used in any way and in any combination (Glaser, 2008; Glaser & Strauss, 1967). However, in accordance with theoretical sampling, data must earn their way into the analysis based on preliminary hypotheses emerging during the process of generating the theory. How the different sources of data earned their way into the analysis is further described in the data collection section.

This process of theoretical sampling was performed in the selective and theoretical phase of the data and analysis process with the purpose of saturating the emerging theory.

#### Memos

Theoretical memos (theoretical notes about the data, concepts, and conceptualization process) played a vital role in generating and articulating the theory in the last and theoretical phase (Glaser, 1978). Writing memos paced the process of theory generation, helped store ideas and make them sortable, and forced us to verify categories and avoid premature conclusions. Memos became the primary source for saturating the categories and determining their interrelationship.

### Sampling procedure and patients

Fifteen patients agreed to participate in the study. One patient (Participant A) was subsequently excluded because her operation was postponed due to a skin lesion in the surgical area. Consequently, 14 patients (eight women and six men) were recruited to this GT study (Table I).

**Table I:** The patients’ sociodemographic and clinical characteristics (*n*=14).

Patient ID	Coping type	Sex	Age (years)	BMI	Level of education^a^	Living with spouse	Primary healthcare service	Years since diagnosis	Number of comorbidities	Anxiety preop/post-op/pre-dis (VAS-anxiety)	Self-rated health preop/post-op (EQ-5D-VAS)
B	Chal	F	65	17.7	3	Yes	No	10	0	5/23/5	90/95
C	Chal	F	78	23.1	3	No	No	Unknown	1	48/30/14	80/100
D	Prot	F	77	30.5	3	Yes	No	Unknown	1	39/–/24	70/84
E	Prot	F	59	38.1	4	No	No	Unknown	0	73/81/18	81/90
F	Chal	M	53	32.8	3	Yes	No	5	1	25/1/3	94/94
G	Acc	M	67	26.3	3	Yes	No	2	3	7/14/13	51/92
H	Prot	F	70	27.6	3	Yes	No	4	0	100/100/17	83/90
I	Acc	M	61	26.9	1	Yes	No	Unknown	0	17/23/3	78/–
J	Exc	F	59	41.5	4	Yes	No	3	3	–/83/46	79/–
K	Chal	M	53	33.2	3	Yes	No	Unknown	1	–/5/1	20/58
L	Exc	F	56	30.5	2	Yes	No	1	1	8/2/1	49/93
M	Acc	M	69	24	4	Yes	No	Unknown	1	21/5/3	–/88
N	Exc	M	58	30.6	3	Yes	No	Unknown	1	30/27/15	95/–
O	Exc	F	52	35.4	2	Yes	No	Unknown	3	−/0/0	38/90

a Stage 1: Primary education/first stage of basic education; Stage 2: second stage of basic education; Stage 3: (upper) secondary education (International Standard Classification of Education (www.unesco.org/education/information/nfsunesco/doc/isced_1997.htm). ID, identification; exc, exceeding boundaries of capability; prot, protecting boundaries of capability; chal, challenging boundaries of capability; acc, accepting boundaries of capability; F, female; M, male; BMI, body mass index; preop, preoperative; post-op, post-operative; pre-dis, pre-discharge; dash indicates missing data; VAS-anxiety, visual analogue scale for anxiety; EQ-5D, EuroQol five-dimension questionnaire.

In cooperation with a nurse in the outpatient clinic, a research assistant conducted inclusion of the 15 patients referred for fast track THA. Prior to inclusion in the study, participants were orally informed about the study as well as their rights as study patients. They all gave oral consent to participation in the outpatient clinic and written consent when attending the information meeting 1–2 weeks prior hospitalization. The general inclusion criteria were patients undergoing their first THA surgery. Exclusion criteria were previous THA surgery, as previous experiences with surgery may impact coping behaviour; acquired or congenital cognitive deficiency; and inability to speak or read Danish.

#### Description of the regional fast track THA programme

The study patients joined a fast track THA programme grounded on a specific fast track concept (Husted et al., 2010). In general, the time from setting the surgery appointment (in the outpatient clinic) to the day of surgery was approximately 2–3 weeks. The THA was converted to same-day admission followed by an in-hospital LOS of 1 day, reducing the time available to prepare the patient for surgery, discharge, and rehabilitation. The reduced time for preparation presupposes profound and extensive information on the programme and the tasks expected (Husted et al., 2012). The standard programme for undergoing fast track THA is described to the patient first at the outpatient clinic by the healthcare professionals. Subsequently, a 36-page patient pamphlet is given to the patients to read before attending the information meeting. Patients and relatives are then invited to a 2-h group informational meeting approximately 1–2 weeks before the operation. Pivotal to this meeting is the presentation of a clear division of pre- and post-operative tasks between the healthcare professionals and patients. Healthcare professionals continuously articulate this division of tasks during the hospital stay.

**Figure 1:**
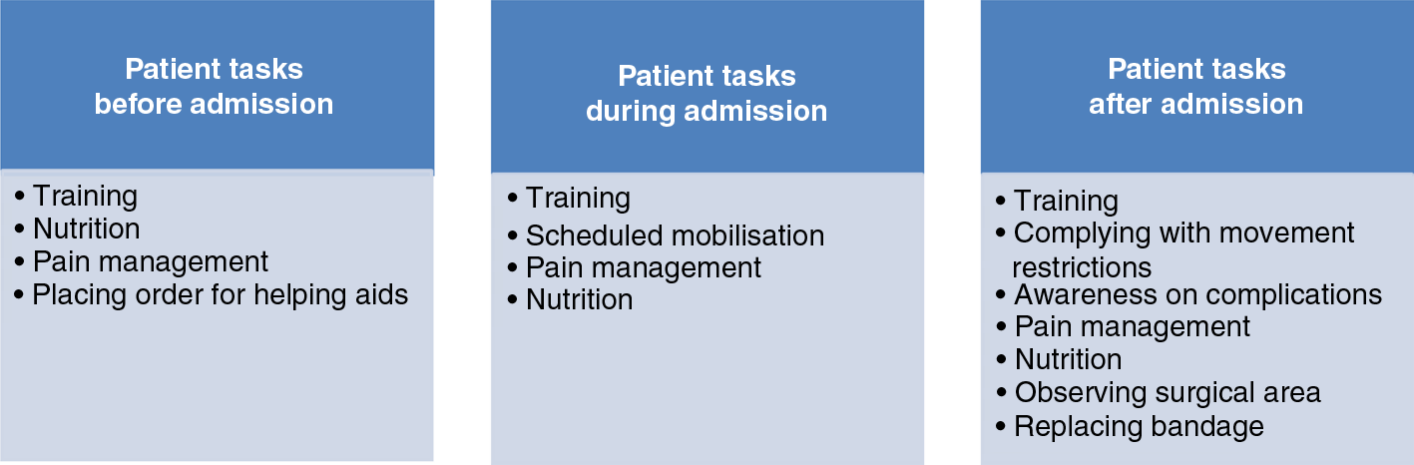
Patient tasks during a fast track total hip replacement programme.

As illustrated in Figure 1, the patients must pre- and post-operatively take responsibility for several tasks before and during admission and after discharge. Training is underscored to the patients as one of the most pivotal tasks, meaning compliance with an exercise programme containing a variety of evidence-based exercises chosen by the team of physiotherapists.

Throughout the programme, starting on the day at the outpatient clinic when surgery was first scheduled and ending 3 weeks after discharge, patients are recommended to contact the coordinator responsible for continuity of care (a specially trained nurse or physiotherapist) if they have questions or doubts concerning their well-being or the programme. The day after discharge, the coordinator phones the patients with the purpose of monitoring their well-being and, during the third post-operative week, patients have an appointment with a physiotherapist in the outpatient clinic for a final follow-up on their functional level and well-being.

### Data collection

**Figure 2:**
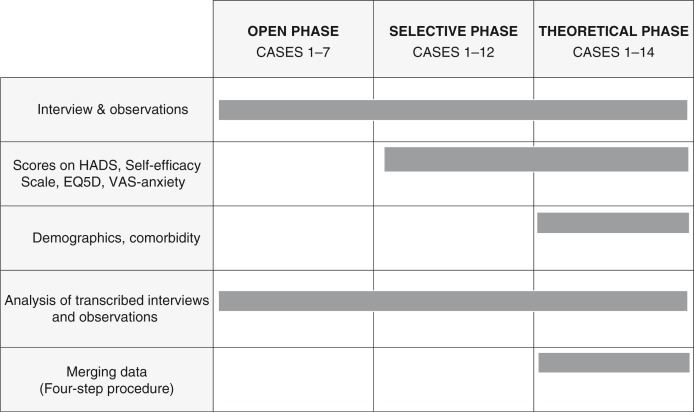
Chronology of data collection and analysis processes in grounded theory. HADS, hospital anxiety and depression scale; EQ-5D, EuroQol five-dimension questionnaire; VAS, visual analogue scale.

Data were iteratively collected at the hospital. The total database consisted of both pre-planned and new data. Figure 2 illustrates the data sources and the chronology for data collecting and analysis.

The pre-planned data were chosen because these data were expected to contain information on the cognitive and behavioural dimensions of coping.

#### Pre-planned data: observations and interviews

Aiming to explain the interacting cognitive and behavioural dimensions of coping, we chose ethnographic methods combining participant observation and semi-structured interviews. These was the pre-planned data-sources and methods. The foci of observations covered the different steps in the fast track THA programme and included data on patient activities and interactions with healthcare professionals during admission. Observations were documented in field notes and used as a basis for interviewing the patients before discharge. The patients’ reflections, views, and experiences were included via interviews (Hammersley & Atkinson, 1996). Guides were used to conduct the observations and interviews and all observations and interviews were transcribed according to a transcriptional guide.

#### New data

Data on the participants' psychological profile derived from validated questionnnaires and socio-demographic. In classical GT, hypotheses generated through the iterative process of theoretical sampling decide the next data to be collected (Glaser & Strauss, 1967). Thus, the pre-planned data sources (patient behaviour and patient narration) were the foundation for collecting additional data. Additional data on the patients were collected during a sub-study, but the data were not known to the researcher before conducting observations and interviews in the open phase. The sub-study aimed to describe the psychological profile of patients going through a THA programme due to the claim that very few studies have identified the patient characteristics that influence the pre- and post-operative process of fast track THA programmes (Husted, Lunn, Troelsen, Gaarn-Larsen, Kristensen, & Kehlet, 2011). The argument for including these new data on the psychological profile were grounded in the pre-planned data hypothesizing that the patients struggled with anxiety, low trust in one's own capability, obesity, information overload and low life quality in coping with the programme.

Consequently data on health outcome, self-efficacy, and anxiety and depression before admission and 3 months after discharge were included for the purpose of identifying coping type-specific features using the following validated questionnaires: EQ-5D-3L (EuroQol five-dimension questionnaire) (Brooks, 1996; EuroQol Group, 1990), Self-Efficacy Scale (Schwarzer & Jerusalem, 1995), and the Hospital Anxiety and Depression Scale (Herman, 1997). Finally, LOS and visual analogue scale for anxiety (VAS-anxiety) (Cella & Perry, 1986) measured at the anaesthetic outpatient clinic just before going to the operating theatre and immediately before discharge were included.

The following data sources were found valid for explaining coping behaviour among patients undergoing THA:

1. Level of education was included in the analysis because coping behaviour characterised by nervousness related to the THA programme had the average higheste education level measured using UNESCO's classification system.
2. Body mass index was included in the analysis because obesity appeared in two of the identified coping types, suggesting a relationship between coping type, stigmatization, and self-blame.
3. The level of VAS-anxiety measured in the anaesthetic outpatient clinic and level of self-rated health measured on the EQ-5D-VAS seemed to be able to discriminate between the identified coping types. Consequently, these scores were also included in the analysis.

### Data analysis

**Figure 3:**
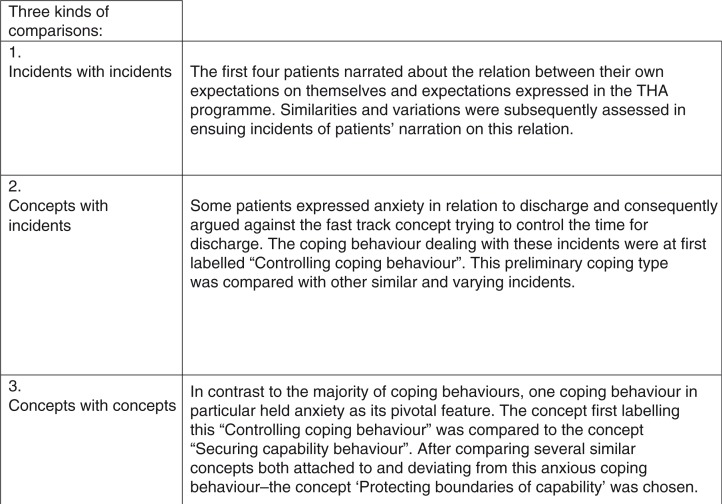
Exemplification of the constant comparison procedure.

The constant comparison procedure of GT was conducted throughout the open, selective, and theoretical phase of the joint data, coding, and analysis process (Glaser, 2001). Three kinds of comparisons were performed. First, incidents were compared to incidents with a focus on similarities and variation, generating concepts and hypotheses. Second, concepts were compared to more incidents to theorize, saturate, and verify concepts in order to generate further concepts. Third, concepts were compared to concepts to determine the most fitting set of concepts and indicators constituting the theory. Figure 3 shows the timeline for these three different kinds of comparisons in the constant comparative process.

The joint data collection and analysis produced two kinds of subordinated analysis processes embraced in the constant comparison procedure. First, transcribed observational and interview data were analysed, focusing on identifying and conceptualizing the possible interplay of patients’ thoughts, feelings, and behaviour when coping with THA. Four preliminary coping types emerged through this analysis. Second, in order to further identify possible coping type-specific features and finally saturate the preliminary theory on the four coping types, qualitative and quantitative data were merged in three chronological steps: (1) patients were listed according to their type of coping behaviour grounded on observations and interviews; (2) the new data were incorporated into an Excel spreadsheet; (3) in order to identify coping type-specific features we searched for features with the ability to discriminate between the four coping types.

### Ethical considerations

The Danish Data Protection Agency gave permission to conduct the study (J. No. 2007-58-0010). According to the Scientific Committee for the County of Central Jutland, the Biomedical Research Ethics Committee System Act did not apply to the study. With reference to the Helsinki Declaration (World Medical Association Declaration of Helsinki, 2000), all patients were informed both verbally and in writing about participation in the study, specifically, that they could at any stage withdraw from participation without compromising their care.

## Results

The main concern among patients coping with total hip replacement in a fast track THA programme is to restore their physical and psychosocial integrity, which has been compromised by a low level of function and mobility in daily life. They cope predominantly by economizing their mental resources, striving to fulfil the expectations of the fast track programme, which expresses the need for patients to be mentally proactive and physically active. In this way they aim to regain their habitual level of function and mobility and, subsequently, their social life and self-image as active human beings.

**Figure 4:**
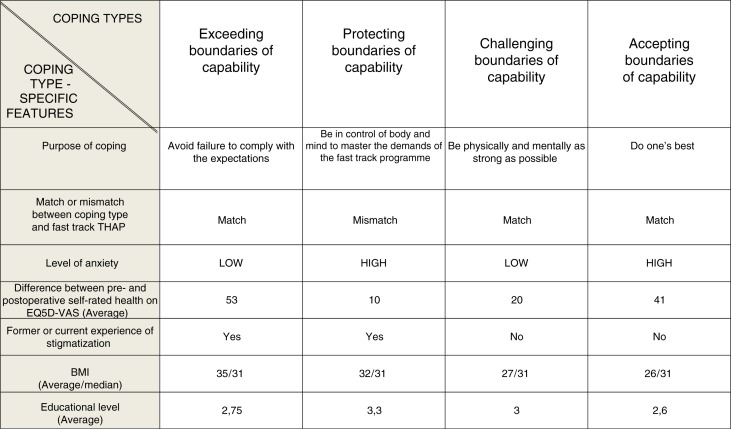
Coping type-specific features. THAP, total hip arthroplasty programme; AOC, anaesthetic outpatient clinic; low degree of anxiety: ≤30 mm on VAS-anxiety; high degree of anxiety: >30 mm on VAS-anxiety; BMI, body mass index; educational level: Stage 1, primary education/first stage of basic education; Stage 2, second stage of basic education; Stage 3, (upper) secondary education (International Standard Classification of Education) (www.unesco.org/education/information/nfsunesco/doc/isced_1997.htm).

Four types of coping behaviour were identified and labelled as follows: exceeding the boundaries of capability, protecting the boundaries of capability, challenging the boundaries of capability, and accepting the boundaries of capability. These predominant coping types had discriminating purposes and other physiological, cognitive, affective, and psychosocial coping-type-specific features (Figure 4).

**Figure 5:**
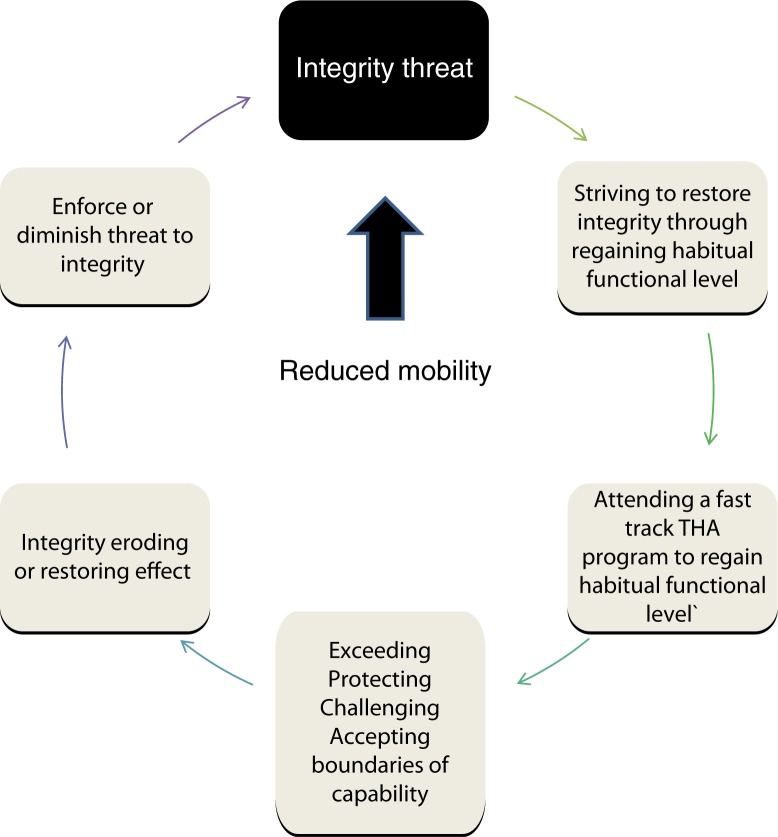
The process of coping with a fast track total hip arthroplasty programme.

As shown in Figure 5, coping is a circular process that causes either an eroding or restoring effect on integrity, depending on whether the coping behaviour matches the expectations expressed in the fast track THA programme.

Across the four coping types, sharing knowledge with fellow patients and relying on support from relatives were important strategies in patients’ efforts to restore their physical and psychosocial integrity. Several other strategies appeared with the aim of economizing both mental and physical resources. For the patients, economizing meant administering resources in an efficient and suitable manner so they would have the strength to restore their integrity by regaining their habitual level of function and self-image. The economizing strategies are illustrated in Tables II and III.

**Table II:** Strategies to economize mental and physical resources during the fast track THA programme.

	Striving to follow the fast track programme	Gaining as much knowledge on how to recover	Planning proactively for recovery at home	Expressing belief in positive reinforcement regarding exercise capacity	Striving for discharge	Consciously suppressing anxiety due to exercise
Exceeding boundaries of capability	***√***	***√***	***√***		***√***	
Protecting boundaries of capability	***√***	***√***	***√***			
Challenging boundaries of capability	***√***	***√***	***√***	***√***	***√***	***√***
Accepting boundaries of capability	***√***	***√***	***√***	***√***		***√***

**Table III:** Strategies to economize mental and physical resources during the fast track THA programme.

	Claiming the right not to follow the fast track programme	Using distraction to control pain and anxiety	Expressing belief in positive reinforcement regarding exercise capacity	Curbing feelings and thoughts	Reducing disruptions by healthcare professionals	Nurturing relations with healthcare professionals
Exceeding boundaries of capability		***√***		***√***	***√***	***√***
Protecting boundaries of capability	***√***	***√***		***√***		***√***
Challenging boundaries of capability		***√***	***√***	***√***		***√***
Accepting boundaries of capability		***√***	***√***	***√***	***√***	***√***

### A common issue among all coping types: To match the THA programme

A common wish for all patients across coping types was to match the standard programme for THA and the subsequent plan for rehabilitation. From the patients’ point of view, complying with the scheduled rehabilitation meant accomplishing the following tasks: (1) early post-operative mobilization; (2) following the scheduled plan for continuous exercise during the hospital stay; (3) finding a balance between exercise and rest; (4) sufficient pain management; (5) sufficient nutrition; and (6) early discharge on the first post-operative day, if possible. Thus, all patients stressed the importance of taking responsibility for their own recovery, which was achieved by following the plan. Not deviating from the original recovery plan meant that the patients themselves enhanced their chances of regaining their habitual physical capacity and minimizing the risk of complications.

The patients focused on the degree to which their effort to accomplish the tasks matched the expectations of the programme. Their narration and behaviour had three cardinal pivots: the purpose for complying with expectations, the boundaries of their capability to comply with the tasks, and how and to what degree the expectations of the program matched the boundaries of their capability.

### Four types of coping with the multimodal fast track THA programme

#### Exceeding the boundaries of capability

The most prominent purpose of exceeding the boundaries of capability was actually to avoid not complying with the expectations. Patients with this coping behaviour exceeded the boundaries of their capability by over-exercising and having difficulty finding a balance between exercise and rest.

I will manage the challenges myself … I may cross a line with what I'm capable of doing.

Sometimes the healthcare professionals corrected overexerting behaviour among patients with this coping behaviour, as they were not cautious enough about risking their new hip. Instead, they were more nervous about not being able to live up to expectations. Consequently, not following the scheduled rehabilitation put pressure on the patients. This occurred for two reasons: First and foremost, following the scheduled exercise program was a prerequisite for them to have the chance to regain their habitual physical level. Second, the patients needed to accomplish the assigned tasks and express pride in performing well for the sake of “good performance.”

I was out of my bed in the recovery ward … the nurses and doctors were wildly impressed with me. To do my best is so important…

By this behaviour they strived not to deviate from the norm of being a patient in a THA programme, compensating for earlier experiences of being bullied as adults or feeling guilt and being stigmatized due to obesity. Patients with this coping behaviour experienced defeat and felt annoyed if they failed to follow the scheduled exercise plan.

#### Protecting the boundaries of capability

Patients who demonstrated protective behaviour towards the boundaries of their capability were driven by a need to be in control of their body and mind and to take charge of the demands of the fast track THA programme. This drive was of existential importance to this group of patients, who appeared to have a profound fear of complications during and after the operation, citing “Murphy's law” or stating “what can go wrong will go wrong.”

Patients with this coping behaviour were especially nervous about discharge and the recovery period at home, and they feared and sometimes resented being responsible for their own recovery. This behaviour was especially evident when the healthcare professionals stressed this responsibility. Consequently, the patients generally reacted with counter pressure, claiming their right to protect their boundaries of capability and to not comply with expectations.

If they (healthcare professionals) only knew … what pressure does to me … I'm not able to push myself more than I already do … they should give me a carrot instead of the stick. (E)

These patients seemed to challenge the healthcare professionals. Consequently, patients with this coping preference initiated a power struggle with healthcare professionals in relation to mobilization, nutrition, and pain management.

Healthcare professionals assessed patients with this coping behaviour to forget the importance of challenging their own boundaries of capability during the rehabilitation process. Similarly, the patients described themselves as “critical” and expressed an understanding that they might be labelled as “difficult patients” by not complying with the standard expectations. Nonetheless, they also felt misunderstood and their boundaries ignored, escalating the power struggle and leaving them frustrated, angry, and sad.

Patients with this coping behaviour were the most well educated, the most anxious patients, and the group with the smallest positive difference in health status 3 months after surgery. They sought additional information to the information they were offered by the healthcare system, and nevertheless or as a consequence they expressed difficulties with maintaining a good grasp of pivotal information and tasks in the post-operative phase. Their preoperative anxiousness drove them to seek information pre-admission.

#### Challenging the boundaries of capability

The purpose of challenging the boundaries of capability and striving to regain physical and mental strength is to be physically and mentally strong. Patients with this type of coping behaviour had previous experience with sports and sports competition, and they enjoyed competing and comparing themselves with fellow patients. These patients were active up until their surgery despite suffering from pain and reduced mobility. However, the stimulus for the surgery was a discontent with their level of activity and consequently a compromised self-image as an active person. After surgery, the patients continuously challenged their physical boundaries in order to monitor and assess the status of their recovery process. The assigned tasks were not present as a source of pressure for patients with this coping behaviour. On the contrary, the tasks appealed to the patients’ competitive and risk-taking behaviour. One of the patients stated:

I couldn't wait to test my new hip … you have to test it sooner or later … so why not immediately…

Patients with this coping behaviour were determined to regain their habitual level of function by transforming unused time to time for exercise:

I couldn't sleep last night … so why not use the time to exercise …

Despite challenging their boundaries, these patients managed to reach a balance between exercise and rest, and they were well prepared and well informed about their assigned tasks. Thus, these patients welcomed the responsibility for their own recovery, and the conviction that the fast track programme was necessary for recovery appeared to be predominant in this type of coping behaviour. Achieving the goal of being discharged on the first post-operative day was a pivotal sign of recovery. The patients were not nervous about being discharged. Instead, they highlighted their plans for rehabilitation at home and focused on their goal of being an active person again, rejoining their family and social network.

#### Accepting the boundaries of capability

Patients who accepted their boundaries of capability as a means to cope with the fast track THA programme possessed an awareness of their capability and relied on this insight despite the expectations of the fast track programme.

I decide for myself what I can accomplish … and you know yourself what you are able to do and what you cannot do …

It's sometimes better to know your limits than to know your achievements …

These patients did not strive to comply with the expectations of being active in the rehabilitation process, and they did not feel pressured by the expected responsibility for their own recovery. They aimed to do the best they could and seemed to comply with the expectations anyway. Taking breaks and taking their time are consistent features of this coping behaviour as well as finding the level of information sufficient and not being anxious about discharge.

The patients managed to economize their physical and mental capabilities in order to have resources to regain their physical strength after surgery. Patients with this coping behaviour had the highest frequency of chronic comorbidities and consequently physical challenges. However, they seemed to cope with this by stressing the importance of “positive reinforcement” in their effort to recover:

You have to think positively … and have faith in the process of getting back to normal…

I used to say … you can do twice as much as you think yourself and ten times more than your mother thinks…

In summary, four coping types were identified with distinct purposes and other physiological, cognitive, affective, and psychosocial coping type-specific features. The main concern among these patients was to regain their habitual level of function, thereby restoring their physical and psychosocial integrity.

## Discussion

The multimodal GT approach generated a theory that revealed four predominant coping types with discriminating purposes and features. We have not found any studies that explore coping with the same aim, design, and methods. Furthermore, a literature search found no studies on predominant coping types in the setting of fast track THA. Coping research is far more disseminated in the area of chronic diseases (Lacasse, Goldstein, Lasserson & Martin, 2006), but the heterogeneity of coping research and coping literature challenges the application of the findings from one setting to another (Skinner, Edge, Altman, & Sherwood, 2003).

Impeded function in daily life compromises the integrity of chronically ill patients (Jørgensen, Dahl, Pedersen & Lomborg, 2013b; Morse, 1997). The current GT highlights this effect on integrity in patients coping with a fast track THA programme. All patients seem to strive to protect their integrity, but in different ways. First, THA patients are compromised in their mobility, often having a healthy body with the physical capacity to rehabilitate to their former physical condition and daily life, as opposed to during chronic illness (Jørgensen et al., 2013b, 2014). The patients, regardless of coping preferences, expect that the THA will give them the opportunity to continue their previously active life. On the other hand, chronically ill pulmonary patients are, to a large extent, permanently compromised regarding their physical capacity in daily life (Jørgensen et al., 2013b). This difference may explain why patients undergoing THA seem to aim to *restore* their integrity by regaining their former physical capacity, whereas pulmonary patients *preserve* their integrity by trying to maintain their current compromised capacity because they know that recovery is not an option (Jørgensen et al., 2013b). This is important knowledge on how different states of physical capacity influence the coping behaviour, forming the basis for individualizing and differentiating patient support.

Fast track THA reduces morbidity, mortality, and functional convalescence (Husted et al., 2012). Consequently, patients meeting the demands of a fast track programme are expected to have a lower risk of complications and a rapid recovery, which also appeared to be pivotal goals for restoring integrity among the patients. The current theory is that all patients in a fast track THA programme strive to meet the education, mental, and physical demands, and the majority are successful. Regardless of coping preferences, patients attributed their recovery to their effort to meet expectations set by the fast track programme. Successfully accomplishing the programme meant an increased chance of recovery, thereby restoring their integrity.

Husted et al. (2012) stressed the necessity of educating healthcare staff to have a uniform approach toward the fast track programme and the patients involved. A uniform approach is intended to produce a consistent programme with clear expectations for the patients, achieving the maximum benefit of the THA without complications (Husted et al., 2012). This clarity in the division of tasks between patients, relatives, and healthcare professionals was also appreciated among the patients, regardless of coping preferences. However, the coping behaviour that did not match the THA (*protecting the boundaries of capability*) seemed to result in opposition to the uniform approach if patients felt pushed to meet the fast track expectations. This finding suggests that patients with certain preferences may require an alternative version of the fast track programme or tailored support in managing the programme.

The most consistent purpose in coping with a fast track THA seems to be existential, expressed as a need to restore physical and psychosocial integrity. However, each coping type has its own subordinate purpose for coping with the programme. These purposes offer the opportunity for healthcare staff to understand what is at stake in each coping type. Patients who exceed or protect their boundaries of capability may compromise the rehabilitation process by overstraining either their physical or mental capacity. Thus, the coping type-specific purposes and features constituting coping types or preferences may create a foundation for identifying more vulnerable patients and offering individualized support, as recommended in previous studies (Norlyk & Harder, 2009). The average lowest increase in self-reported health status was found in the most anxious patients, who exhibited protective behaviour towards their capability boundaries. This finding is in line with a study finding that psychological distress may be a predictor of poor treatment outcome (Howard, Ellis & Khaleel, 2010). Distress may not be fast track–initiated, as major depression and anxiety disorder are relatively common among patients in fast track knee and hip arthroplasty programmes (Riddle, Wade & Jiranek, 2010). However, these common disorders call for identifying the most vulnerable patients and offering them alternative education and psychological support as they go through a fast track orthopaedic programme.

The healthcare system is obliged to secure the conditions for empowering patients to develop the necessary skills, confidence, and knowledge to be active partners in their own healthcare (Lancet Editorial, 2005). None of the patients objected to the expectation of being active. However, the confidence to accomplish a fast track THA programme was lacking in the most anxious patients, who protected their boundaries of capability. These patients did experience a mental and education overload when attempting to gain control and minimize anxiety by increasing their knowledge of the programme. We know that extensive preoperative information enhances patient knowledge, satisfaction, and health-related quality of life; reduces preoperative anxiety; improves peri-operative anaesthesia; and reduces post-operative use of analgesic (Husted et al., 2012; Rolfson, Dahlberg, Nilsson, Malchau, & Garellick, 2009; Sjöling, Nordahl, Olofsson, & Asplund, 2003). However, this extensive information should be tailored to the patient. The current theory states that the most anxious patients and those overloaded with information (protecting the boundaries of their capability) may benefit from individualized information.

Tailored information can be achieved by taking health literacy into account. Strengthening patients’ health literacy is a pivotal element in empowering them (Lancet Editorial, 2005) because health literacy correlates positively with improved learning abilities, low mortality rate (Bostock & Steptoe, 2012), and a high level of self-efficacy related to accomplishing preoperative, peri-operative, and post-operative tasks surrounding orthopaedic surgical procedures (Pellino et al., 1998; Salmon & Hall, 2003).

### Assessing the quality of the GT

The quality of a generated theory depends on how fit, workable, relevant, and modifiable the theory is, as these are the interacting criteria for evaluating the quality of a GT (Glaser, 1978). The theory must *fit* the data; *work* to explain, predict, and interpret the studied empirical area; be *relevant*, meaning that the theory must focus on the main concern of the people under study; and must be *modifiable* with contextual changes (Glaser, 1978; Glaser & Strauss, 1967). We abstained from using preconceived categories or theories and stringently conducted the joint data, coding, and analysis procedure with the guidance of the theoretical sampling process, deductively testing and inductively initiating hypotheses. This approach should meet the four criteria (Glaser, 1978).

Glaser and Strauss emphasized that transparency is not needed for the sake of verifying the theory, but for the sake of giving peers the opportunity to judge the credibility of the theory (Glaser & Strauss, 1967). We argue that the criteria for judging the theory should be based on details about the actual strategy used for collecting, coding, analysing, and presenting the data when generating the theory in accordance with Glaser and Strauss (1967). We strived to meet these criteria, conveying the research strategy used for generating this GT on how patients cope with a fast track THA programme.

## Conclusion

In theory, four predominant types of coping with discriminating purposes and physiological, cognitive, affective, and psychosocial features were observed among patients in a fast track THA programme. Regardless of coping preferences, all patients appraised the fast track programme as necessary for recovery and restoring their physical and psychosocial integrity. One out of the four coping types did not match the expectations of the fast track programme, resulting in eroded integrity. The mismatch seemed to be due, in part, to anxiousness and information overload. Although the fast track concept highlights the necessity of a uniform approach to facilitate the patients’ participation in a fast track programme, coping preferences may require an individualized approach, for example by providing tailored information taking the patient's degree of health literacy into account. Such consideration may offer better conditions for empowering patients to meet the educational, physical, and mental demands of a fast track THA programme.

## Relevance to clinical practice

Coping preferences with coping type-specific features may create the opportunity to enhance our knowledge of the physiological, cognitive, affective, and psychosocial mechanisms involved in successfully dealing with a fast track programme. The most clinically important features discriminate the non-matching coping type (*protecting the boundaries of capability*) from the three remaining coping types, which enable patients to meet the fast track expectations. Thus, the generated theory may form the basis for identifying individual preferences for coping and offer tailored support for optimizing self-management competencies.

However, clinical application of a theory on coping calls for ethical considerations. Coping literature *per se* may impede patients’ coping skills. In addition, healthcare professionals’ use of concepts and pre-understanding derived from theories and predefined labels can influence patient identity, capability, and coping behaviour (Telford, Kralik & Koch, 2006).

The theory on coping preferences and coping type-specific features could be deductively tested with a greater number of patients and challenged by new data in this area of research.

## Conflict of interest and funding

The authors have not received any funding or benefits from industry or elsewhere to conduct this study.
